# A new synonym of a species of *Stachyotropha* Stål, 1871, a genus of Asian Stenopodainae (Hemiptera, Heteroptera, Reduviidae)

**DOI:** 10.3897/BDJ.11.e102977

**Published:** 2023-04-11

**Authors:** Kyosuke Okuda, Zhuo Chen

**Affiliations:** 1 CTI REED Co., Ltd., Kamikisaki1-14-6, Urawa-ku, Saitama-shi, Saitama, 330-0071, Japan CTI REED Co., Ltd. Kamikisaki1-14-6, Urawa-ku, Saitama-shi, Saitama, 330-0071 Japan; 2 Saitama Museum of National History (External researcher: Animal), Nagatoro 1417-1, Nagatoro, Saitama, 369-1305, Japan Saitama Museum of National History (External researcher: Animal) Nagatoro 1417-1, Nagatoro, Saitama, 369-1305 Japan; 3 Department of Entomology and MOA Key Lab of Pest Monitoring and Green Management, College of Plant Protection, China Agricultural University, Yuanmingyuan West Road, Beijing 100193, China Department of Entomology and MOA Key Lab of Pest Monitoring and Green Management, College of Plant Protection, China Agricultural University, Yuanmingyuan West Road Beijing 100193 China

**Keywords:** China, East Asia, Japan, new synonym, Philippines, taxonomy

## Abstract

**Background:**

*Stachyotropha* Stål, 1871 (Reduviidae, Stenopodainae) currently includes only two species: *S.punctifera* Stål, 1871 and *S.miyamotoi* Hidaka & Miller, 1959 only recorded from East Asia.

**New information:**

This study reviews the genus *Stachyotropha* Stål, 1871 and its two described species. Based on the examination of the type specimens and the original descriptions, *S.miyamotoi* Hidaka & Miller, 1959 is regarded as a junior synonym of *S.punctifera*. Further, we briefly discuss the distribution and biology of *S.punctifera*.

## Introduction

Stenopodainae Amyot & Serville, 1843 is the fifth largest subfamily of Reduviidae, with approximately 115 described genera and 720 described species. Most of these species occur in the Tropics (Maldonado Capriles 1990, Ishikawa and Miyamoto 2012, Schuh and Weirauch 2020). The Neotropical and Afrotropical stenopodainae fauna have been relatively well surveyed (Villiers 1948, Villiers 1968, Gil-Santana et al. 2015). Additionally, Miller published voluminous literature about African and Asian faunas (e.g. Miller (1940), Miller (1948), Miller (1950), Miller (1954)); however, half of the Asian fauna remain understudied. Chen et al. (2020) proposed that comprehensive taxonomic revisions of Asian Stenopodainae are needed because the studies before 1970 lacked illustrations and adequate descriptions. Moreover, the taxonomic keys for Asian Stenopodainae are outdated or incomplete now.

One such group is *Stachyotropha* Stål, 1871, which is a genus with only two species, *S.punctifera* Stål, 1871 and *S.miyamotoi* Hidaka & Miller, 1959, that have only been described from East Asia so far (Maldonado Capriles 1990). This genus has unique morphologies and can be distinguished from the other Old-World stenopodainae genera by the following combination of characteristics: the first visible labial segment is longer than the remaining segments combined and the thickened profemur is armed ventrally with two rows of long spines (Ishikawa and Miyamoto 2012). To date, *S.punctifera* has been recorded in the Philippines and southern China, whereas *S.miyamotoi* has only been reported from Japan (China 1940, Hsiao and Ren 1981, Maldonado Capriles 1990, Yasunaga et al. 1993, Ishikawa and Miyamoto 2012, Ishikawa 2016).

Minimal records of species from this genus could have led to an incorrect comparison between the two species in the past. Thus, in this study, we reviewed the genus *Stachyotropha* and its two described species to determine whether *S.punctifera* and *S.miyamotoi* are, indeed, separate species or synonyms of the same species. We also attempted to obtain new biological and distributional information for the genus.

## Materials and methods

Examined type specimens. We examined photos of the types provided by the Swedish Museum of Natural History, Stockholm, Sweden (NHRS). These photos were provided by Gunvi Lindberg and are copyright (2022) of the NHRS. Two male specimens with "Type" and "Paratype" labels were found; however, Stål (1871) did not designate holotype in the original description. Therefore, we treated the two specimens as syntypes.

The holotype of *S.miyamotoi* had been deposited in the Entomological Laboratory, Faculty of Agriculture, Kyushu University, Fukuoka, Japan (ELKU). At the time of the present study, ELKU loaned the holotype to the Entomological Museum of China Agricultural University, Beijing, China (CAU) where we could study it.

Additional specimens. Non-type specimens were obtained from the following institutions: Kanagawa Prefectural Museum of Natural History, Kanagawa, Japan (KPMNH); Laboratory of Entomology, Faculty of Agriculture, Tokyo University of Agriculture, Kanagawa, Japan (ELTUA); Entomological Museum of China Agricultural University, Beijing, China (CAU); and the private collection of Kyosuke Okuda, Saitama, Japan (PCKO).

Specimens collected during recent field surveys by the first author and our colleagues were also studied. Morphological characteristics were observed and measured under a stereomicroscope (Olympus SZ40; Olympus, Tokyo, Japan) equipped with a micrometer. To examine the structure of male genitalia, the male terminalia were soaked in hot 5% potassium hydroxide (KOH) solution for approximately 10 min to remove the tissues. Photographs of the specimens were taken using a single-lens reflex camera (Canon 7D Mark II; Canon, Tokyo, Japan), equipped with a Canon macro lens EF 100 mm and MP-E 65 mm. All morphological terms were assigned in accordance with Weirauch (2008), Ha et al. (2022)and.

## Taxon treatments

### Stachyotropha

Stål, 1871

3DC58D09-40CC-580A-8604-D4335791A4EC

Stachyotropha Stål (1871): 697 (original description); Stål (1874): 84, 85 (in key, catalogue); Lethierry and Severin (1896): 81 (catalogue); China (1940): 210 (in key, fauna of China); Hoffmann (1944): 5 (catalogue, fauna of China); Hsiao (1977): 86 (in key, fauna of China); Hsiao and Ren (1981): 464, 465 (in key, listed, fauna of China); Miyamoto and Yasunaga (1989): 170 (listed, fauna of Japan); Maldonado Capriles (1990): 540 (catalogue); Putshkov and Putshkov (1996): 223 (catalogue, Palearctic); Ishikawa and Miyamoto (2012): 276, 284 (in key, diagnosis, distribution, fauna of Japan); Ishikawa (2016): 450 (listed, fauna of Japan).

#### Description

##### Redescription

**Macropterous male.** Colouration: Basic colouration pale brown with dark marking on pronotum, hemelytra and abdomen.

Vestiture: Head, thorax, abdomen, legs and antennae densely covered with short setae. Compound eyes with sparse short setae. Femur with short decumbent setae; tibia and tarsus with suberect setae.

Structure: Body elongated. Head cylindical, integument granulose; ante-ocular portion longer than postocular portion; lateral side of postocular portion lacking tubercles. Compound eyes spherical, well projected laterad. Ocelli not elevated. Juga (= mandibular plates) strongly produced anteriorly, slightly upwards, slightly shorter than scape (= antennal segment I). Maxillary plates produced anteriorly, straight, approximately half of length of scape. Scape stout, shorter than head; pedicel (= antennal segment II) slender, longer than scape; flagellomeres (= antennal segment III and IV) filiform, shorter than scape. Labium curved; first visible labial segment extend beyond posterior margin of eye, approximately 2 times as long as remaining segments combined.

Pronotum trapezoidal, integument granulose, longer than humeral width and head length; anterior pronotal lobe with four vague glabrous sulci on disc; anterolateral angles obtuse; posterior angles obtuse with posterior margin projecting backwards and slightly concave at mid-portion.

Profemur thickened, with erect setae; lateral inner side armed with three long spines and one or two pairs of short spines, outer side armed with three long spines and one or two pairs of short spines; spines produced slightly downwards and occurring alternately on inner and outer sides. Protibia with two pairs of lateral spines produced slightly downwards and occurring alternately; protarsus three-segmented. Mid-femur and hind femur slender, lacking spines; mid-tarsus and hind tarsus three-segmented; third tarsomere longer than remaining segments.

Abdomen elongate, with lateral margins almost parallel; tergite VII with obliquely angulately truncated inner side, posteromedial margin deeply concave.

**Macropterous female.** Similar to male in general habitus. Abdominal segments II ~ VI with lateral margins almost parallel; segment VII strongly extended backwards, with obliquely angulately truncated inner side, posteromedial margin deeply concave; segment VIII large, transverse; basal half of sternite covered by gonocoxa VIII.

#### Diagnosis

In general appearance, this genus resembles *Campsocnemis* Stål, 1871 known from East Asia, but it can be distinguished from the latter by a combination of the following characters: juga as long as or slightly shorter than scape (in *Campsocnemis*, juga obviously shorter than scape); profemur and protibia armed ventrally with two rows of long diverging spines (in *Campsocnemis*, profemur armed with two rows of finely diverging spines, protibia lacking spines).

#### Diversity

The genus *Stachyotropha* previously contained two species distributed in East Asia (Maldonado Capriles 1990). One of them is treated as a junior synonym in this study; thus, the number of species in this genus has become one.

### Stachyotropha punctifera

Stål, 1871

B566B99E-C7DD-53D7-8FE8-42B3875615C7

Stachyotropha punctifera Stål,1871 - Stål (1871): 698 (original description); Stål (1874): 85 (catalogue, distribution); Lethierry and Severin (1896): 81 (catalogue, distribution); China (1940): 251 (listed); Hoffmann (1944): 5 (catalogue, distribution); Hsiao and Ren (1981): 465 (listeda); Maldonado Capriles (1990): 540 (catalogue, distribution); Putshkov and Putshkov (1996): 223 (catalogue, distribution); Hua (2000): 211 (listed, distribution). Syntypes (2♂): Philippines, NHRS.
Stachyotropha miyamotoi Hidaka and Miller, 1959 - Hidaka and Miller (1959): 136 (original description); Azuma and Kinjo (1987): 36 (catalogue, distribution); Miyamoto and Yasunaga (1989): 170 (listed, distribution); Maldonado Capriles (1990): 540 (catalogue, distribution); Yasunaga et al. (1993): 172 (re-description, distribution, bionomics, photo); Ministry of the Environment Government of Japan (1995): 151 (catalogue); Putshkov and Putshkov (1996): 223 (catalogue, distribution); Koike (1996): 14 (record); Hayashi (2002): 139 (listed); Ishikawa (2005): 30 (photo); Ishikawa and Miyamoto (2012): 284 (distribution, bionomics, photo); Ishikawa (2016): 450 (catalogue, distribution). Holotype (♂): Japan, the Ryukyus, Okinawa Is., ELKU. **New Synonym.**

#### Materials

- **Type status:** Syntype. **Occurrence:** sex: male; lifeStage: adult; occurrenceID: 657FC444-4FA0-54BC-B30A-9C068BF3ACC8; **Taxon:** kingdom: Animalia; phylum: Arthropoda; class: Insecta; order: Hemiptera; family: Reduviidae; genus: Stachyotropha; specificEpithet: *punctifera*; **Location:** country: Philippines; **Identification:** identifiedBy: Stål; dateIdentified: 1871; **Record Level:** institutionID: NHRS; collectionID: GULI-000007698
- **Type status:** Syntype. **Occurrence:** sex: male; lifeStage: adult; occurrenceID: 59815025-84F7-555F-B05F-B503CD92B13D; **Taxon:** kingdom: Animalia; phylum: Arthropoda; class: Insecta; order: Hemiptera; family: Reduviidae; genus: Stachyotropha; specificEpithet: *punctifera*; **Location:** country: Philippines; **Identification:** identifiedBy: Stål; dateIdentified: 1871; **Record Level:** institutionID: NHRS; collectionID: GULI-000001771
- **Type status:** Holotype. **Occurrence:** recordedBy: T. Takara; sex: male; lifeStage: adult; occurrenceID: EC4BBBE8-37FC-595F-B281-6892D628B490; **Taxon:** kingdom: Animalia; phylum: Arthropoda; class: Insecta; order: Hemiptera; family: Reduviidae; genus: Stachyotropha; specificEpithet: *miyamotoi*; **Location:** islandGroup: The Ryukyus; island: Okinawa Is.; country: Japan; stateProvince: Okinawa; **Event:** year: 1955; month: 8; day: 1; **Record Level:** institutionID: ELKU
- **Type status:** Other material. **Occurrence:** recordedBy: Reo Ito; sex: male; lifeStage: adult; occurrenceID: 9F6A721F-EFCF-5824-AB06-8111C3026854; **Taxon:** kingdom: Animalia; phylum: Arthropoda; class: Insecta; order: Hemiptera; family: Reduviidae; genus: Stachyotropha; specificEpithet: *punctifera*; **Location:** islandGroup: The Ryukyus; island: Kakeroma Is.; country: Japan; stateProvince: Kagoshima; county: Ôshima-gun; municipality: Setouchi-chô; locality: Doren; **Identification:** identifiedBy: Kyosuke Okuda; dateIdentified: 2022; **Event:** year: 2022; month: 4; day: 30; **Record Level:** institutionID: PCKO
- **Type status:** Other material. **Occurrence:** recordedBy: Tadafumi Nakata; sex: male; lifeStage: adult; occurrenceID: 47F142CE-D5F9-5983-9904-E4F557CA874C; **Taxon:** kingdom: Animalia; phylum: Arthropoda; class: Insecta; order: Hemiptera; family: Reduviidae; genus: Stachyotropha; specificEpithet: *punctifera*; **Location:** islandGroup: The Ryukyus; island: Ishigaki Is.; country: Japan; stateProvince: Okinawa; municipality: Ishigaki-shi; locality: Omoto; **Identification:** identifiedBy: Kyosuke Okuda; dateIdentified: 2022; **Event:** year: 2003; month: 8; day: 1; **Record Level:** institutionID: PCKO
- **Type status:** Other material. **Occurrence:** recordedBy: Reo Ito; sex: male; lifeStage: adult; occurrenceID: 7AA635D6-C2EB-5EF3-9BE1-80AF4A8D7FCF; **Taxon:** kingdom: Animalia; phylum: Arthropoda; class: Insecta; order: Hemiptera; family: Reduviidae; genus: Stachyotropha; specificEpithet: *punctifera*; **Location:** islandGroup: The Ryukyus; island: Ishigaki Is.; country: Japan; stateProvince: Okinawa; municipality: Ishigaki-shi; locality: Shiraho; **Identification:** identifiedBy: Kyosuke Okuda; dateIdentified: 2022; **Event:** year: 2013; month: 10; day: 7; **Record Level:** institutionID: PCKO
- **Type status:** Other material. **Occurrence:** recordedBy: Kyosuke Okuda; sex: female; lifeStage: adult; occurrenceID: EED80E3C-317A-57AD-A140-57F998577301; **Taxon:** kingdom: Animalia; phylum: Arthropoda; class: Insecta; order: Hemiptera; family: Reduviidae; genus: Stachyotropha; specificEpithet: *punctifera*; **Location:** islandGroup: The Ryukyus; island: Ishigaki Is.; country: Japan; stateProvince: Okinawa; municipality: Ishigaki-shi; locality: Shiraho; **Identification:** identifiedBy: Kyosuke Okuda; dateIdentified: 2022; **Event:** samplingProtocol: light trap; year: 2019; month: 4; day: 20; **Record Level:** institutionID: PCKO
- **Type status:** Other material. **Occurrence:** recordedBy: Tadashi Ishikawa; sex: female; lifeStage: adult; occurrenceID: 72C8F81A-B33A-5A12-AFA3-9929224A4FB2; **Taxon:** kingdom: Animalia; phylum: Arthropoda; class: Insecta; order: Hemiptera; family: Reduviidae; genus: Stachyotropha; specificEpithet: *punctifera*; **Location:** islandGroup: The Ryukyus; island: Iriomote Is.; country: Japan; stateProvince: Okinawa; county: Yaeyama-gun; municipality: Taketomi-chô; locality: Shirahama-rindô; **Identification:** identifiedBy: Kyosuke Okuda; dateIdentified: 2022; **Event:** year: 2003; month: 4; day: 3; **Record Level:** institutionCode: ELTUA
- **Type status:** Other material. **Occurrence:** recordedBy: Yukihiko Hirano; sex: male; lifeStage: adult; occurrenceID: 484306F2-0B40-5E1C-AD86-993A9DA1CD86; **Taxon:** kingdom: Animalia; phylum: Arthropoda; class: Insecta; order: Hemiptera; family: Reduviidae; genus: Stachyotropha; specificEpithet: *punctifera*; **Location:** islandGroup: The Ryukyus; island: Iriomote Is.; country: Japan; stateProvince: Okinawa; county: Yaeyama-gun; municipality: Taketomi-chô; locality: Takana; **Identification:** identifiedBy: Kyosuke Okuda; dateIdentified: 2022; **Event:** year: 2004; month: 4; day: 19; **Record Level:** institutionID: KPMNH
- **Type status:** Other material. **Occurrence:** recordedBy: Kyohei Watanabe; sex: male; lifeStage: adult; occurrenceID: BBDBC5DF-8BBE-50BA-925D-AA37C6684A03; **Taxon:** kingdom: Animalia; phylum: Arthropoda; class: Insecta; order: Hemiptera; family: Reduviidae; genus: Stachyotropha; specificEpithet: *punctifera*; **Location:** islandGroup: The Ryukyus; island: Iriomote Is.; country: Japan; stateProvince: Okinawa; county: Yaeyama-gun; municipality: Taketomi-chô; locality: Ootomi,Haiminaka; **Identification:** identifiedBy: Kyosuke Okuda; dateIdentified: 2022; **Event:** samplingProtocol: light trap; year: 2014; month: 5; day: 15; **Record Level:** institutionCode: KPMNH
- **Type status:** Other material. **Occurrence:** recordedBy: Tomoya Saeki; individualCount: 4; sex: 2males, 2females; lifeStage: adult; occurrenceID: DAE1EC1C-EB63-59AC-B3D0-0CC040EB4A66; **Taxon:** kingdom: Animalia; phylum: Arthropoda; class: Insecta; order: Hemiptera; family: Reduviidae; genus: Stachyotropha; specificEpithet: *punctifera*; **Location:** islandGroup: The Ryukyus; island: Iriomote Is.; country: Japan; stateProvince: Okinawa; county: Yaeyama-gun; municipality: Taketomi-chô; locality: Takana; **Identification:** identifiedBy: Kyosuke Okuda; dateIdentified: 2023; **Event:** samplingProtocol: light trap; year: 2022; month: 5; day: 14; **Record Level:** institutionCode: PCKO
- **Type status:** Other material. **Occurrence:** recordedBy: Chikun Yang; sex: female; lifeStage: adult; occurrenceID: 6D1E6D6D-B155-5F9F-B225-8A5A52BB2A8D; **Taxon:** kingdom: Animalia; phylum: Arthropoda; class: Insecta; order: Hemiptera; family: Reduviidae; genus: Stachyotropha; specificEpithet: *punctifera*; **Location:** country: China; stateProvince: Guangxi; county: Guilin; municipality: Yanshan District; **Identification:** identifiedBy: Kyosuke Okuda and Zhuo Chen; dateIdentified: 2022; **Event:** year: 1963; month: 5; day: 29; **Record Level:** institutionID: CAU; collectionID: RE-0001049
- **Type status:** Other material. **Occurrence:** recordedBy: K. Souma; sex: female; lifeStage: adult; occurrenceID: ACC8DEF0-4674-5D4B-A5C5-297CE5FC957B; **Taxon:** kingdom: Animalia; phylum: Arthropoda; class: Insecta; order: Hemiptera; family: Reduviidae; genus: Stachyotropha; specificEpithet: *punctifera*; **Location:** island: Mindanao Is.; country: Philippines; stateProvince: Davao (Near CALINAN); **Identification:** identifiedBy: Kyosuke Okuda; dateIdentified: 2022; **Event:** year: 1970; month: 6; day: 20; **Record Level:** institutionID: ELTUA

#### Description

##### Redescription

**Figure 1.**
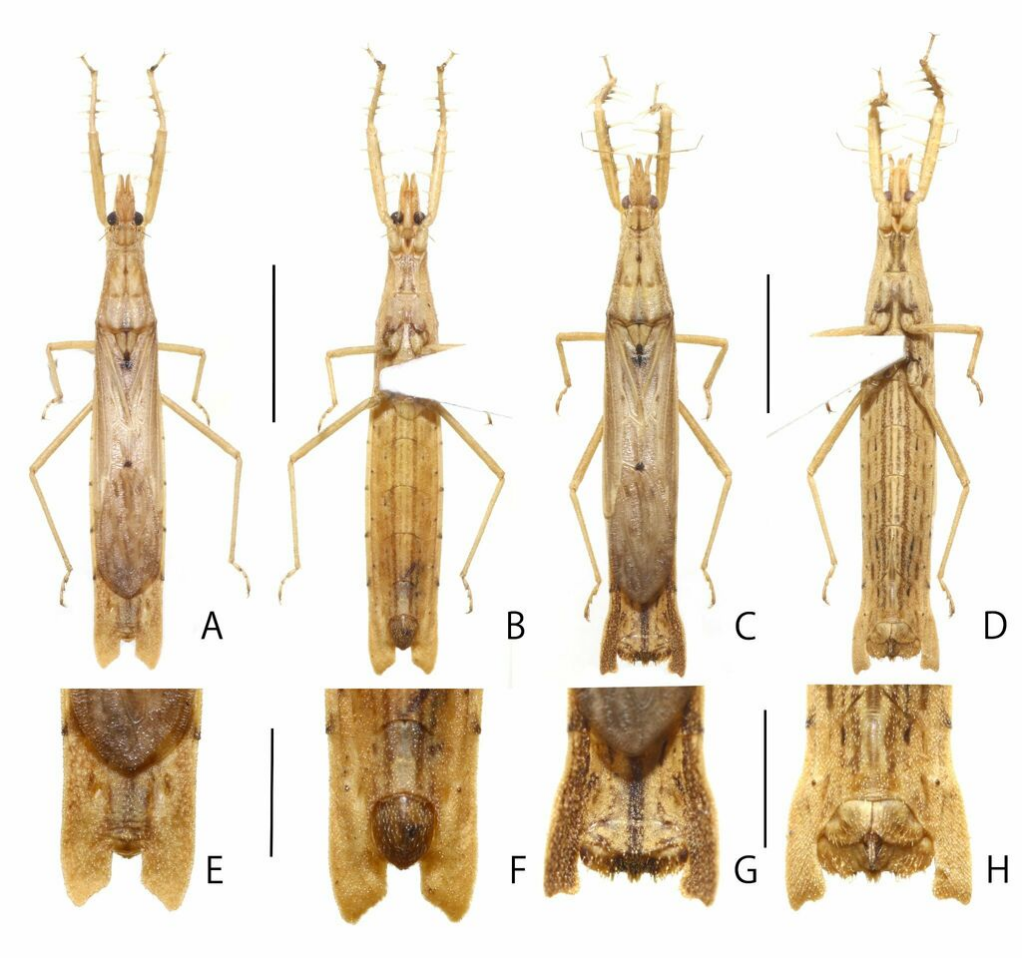
*Stachyotrophapunctifera*: **A, B** male habitus (A: dorsal; B: ventral); **C, D** female habitus (C: dorsal; D: ventral); **E–H** apical segments of abdomen (E: male dorsal; F: male ventral; G: female dorsal; H: female ventral). Scale bars: 5 mm for A–D and 2 mm for E–H.

**Macropterous male.** Colouration: General colour pale yellowish-brown (Fig. 1); body matte, covered with whitish pilose setae on head, thorax, abdomen and legs; setation on corium sparse; eyes and apex of scutellum black; third visible labial segment dark brown; vein of cubitus with blackish spot; connexiva of abdominal tergites III ~ VII with dark spot; abdominal sternites III ~ VII with one pair of longitudinal brownish markings; ventral surface of abdomen with sparse blackish spots.

Vestiture: Head, thorax, abdomen, legs and antennae densely covered with short setae. Scape with short decumbent setae, approximately less than 0.4 times as long as maximum width of scape; segment II ~ IV with long suberect setae, approximately 1.0 ~ 1.3 times as long as maximum width of each antennal segment. Femur with short setae, approximately less than 0.2 times as long as maximum width of femur; tibia with decumbent setae, approximately 0.5 times as long as maximum width of tibia; tarsus with decumbent setae, approximately as long as maximum width of tarsus.

**Figure 2.**
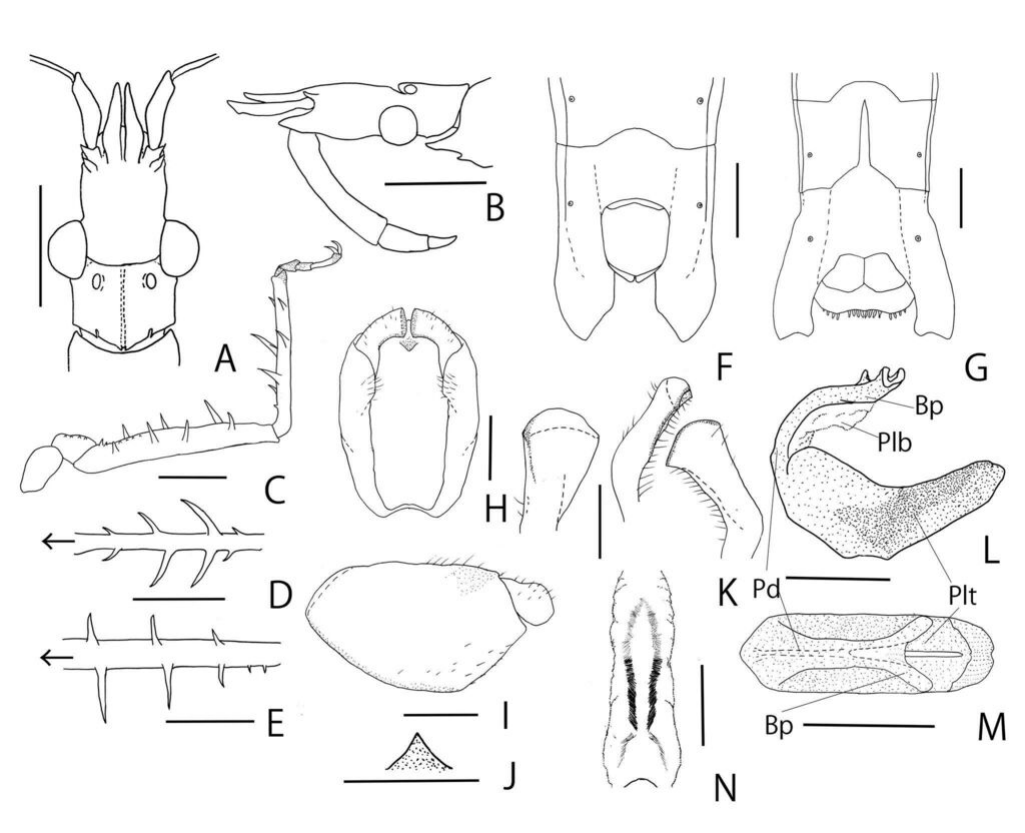
*Stachyotrophapunctifera*: **A, B** head, male (A: dorsal; B: lateral); **C** proleg; **D** protibia, ventral; **E** profemur, ventral; **F, G** abdominal sternite VII, ventral (F: male; G: female); **H, I** pygophore (H: dorsal; I: lateral); **J** median process of pygophore, caudal; **K** paramere, in different views; **L–N** phallus (L: lateral; M: dorsal; N: expanded phallosoma dorsal view). Arrows in D, E indicate front direction. Scale bars: 1 mm for A–G, 0.5 mm for H–I and K–N and 0.25 mm for J. (Abbreviations: bp, basal plates; pd, pedicel; plb, phallobase; plt, phallotheca).

Structure: male: Body medium-sized (16.00 mm), approximately 6.9 times longer than its maximum width (Fig. 1A, B). Head (Fig. 2A) approximately 1.7 times as long as width across eyes, approximately 0.8 times as long as pronotum; ante-ocular portion approximately 1.2 times as long as postocular portion; postocular with dorsal longitional sulcate. Juga approximately 0.85 times as long as scape. Maxillary plates approximately half as long as scape. Scape approximately 0.35 times as long as head; ratio of antennal segments (I ~ IV) = 10.0 : 19.3 : 4.0 : 6.7. First visible labial segment (Fig. 2B) curved, extending to level of middle of eye; ratio of visible labial segments (I ~ III) = 10.0 : 0.31 : 0.20.

Pronotum 1.5 times as long as humeral width; anterior propleural spines thick, acute, curved upwards, shorter than eye width. Hemelytra reaching abdominal segment VII.

Profemur (Fig. 2C, E) with lateral inner side armed three long spines and two pairs short spines; longest spine approximately 1.9 times as long as maximum width of profemur; shortest spines approximately 0.6 times as long as maximum width of profemur. Protibia (Fig. 2D) armed with two pairs of lateral spines; longest spines approximately 3.0 times as long as maximum width of protibia; shortest spines approximately 0.8 times as long as maximum width of protibia.

Abdomen (Fig. 2F) elongate, approximately 3.6 times as long as its maximum width; segment VII with obliquely angulately truncated inner side.

**Male genitalia**. Pygophore (Fig. 2H, I) broadly rounded ventrally, ventral surface covered with short sparse setae; median process of pygophore (Fig. 2J) equilateral triangle from dorsal view, weakly darkened. Paramere (Fig. 2K) thick, apical portion broad, weakly curved, with short erect setae. Phallus (Fig. 2L-N) elongate; dorsal phallothecal sclerite elongate, sclerotised ventrally; basal plate thin from posterior view, entire length approximately 0.6 times as long as dorsal phallothecal sclerite; phallosoma elongate, nearly cylindical; struts nearly straight, not reaching apex of dorsal phallothecal sclerite.

**Macropterous female.** Similar to male in general habitus (Fig. 1C, D). Body (16.5 mm) approximately 4.7 times as long as than its maximum width. Abdomen (Fig. 2G) approximately 3.2 times as long as its maximum width.

#### Distribution

**China**: Fujian, Guangxi; **Japan**: The Ryukyus (Kakeroma Island, Okinawa Island, Miyako Island, Ishigaki Island, Iriomote Island, Yonaguni Island); **Philippines**: Mindanao Island.

#### Biology

In Japan, almost all examined specimens were collected using light traps. This species was very rare everywhere. Ishikawa and Miyamoto (2012) have previously recorded the adults and nymphs (as *S.miyamotoi*) collected from grasslands, where Poaceae and Cyperaceae were dominant. Additionally, adults also have known examples collected from an ankle-deep marshy area.

## Discussion

Stål (1871) described *Stachyotrophapunctifera*, based on an unspecified number of male specimens collected from the Philippines. He provided a dorsal habitus illustration of this species with the original description. Hidaka and Miller (1959) described *Stachyotrophamiyamotoi*, based on one male collected from the Okinawa Island of the Ryukyu Islands and provided the illustrations of the dorsal habitus and the lateral view of the anterior body part. They listed the following morphological characters to distinguish *S.miyamotoi* and *S.punctifera*: 1) the dorsal surface of the head of *S.miyamotoi* is considerably less strongly sulcate; 2) the antennifers of *S.miyamotoi* are obtusely conical; 3) the armature of the prolegs is different between the two species; 4) the apex of the produced portions of abdominal segment VII of *S.miyamotoi* is obliquely, but not angulately truncated; and 5) the apical internal cell of the hemelytron is less than half as long as the apical external cell at the base in *S.miyamotoi*, whereas it is half as long in *S.punctifera*.

**Figure 3.**
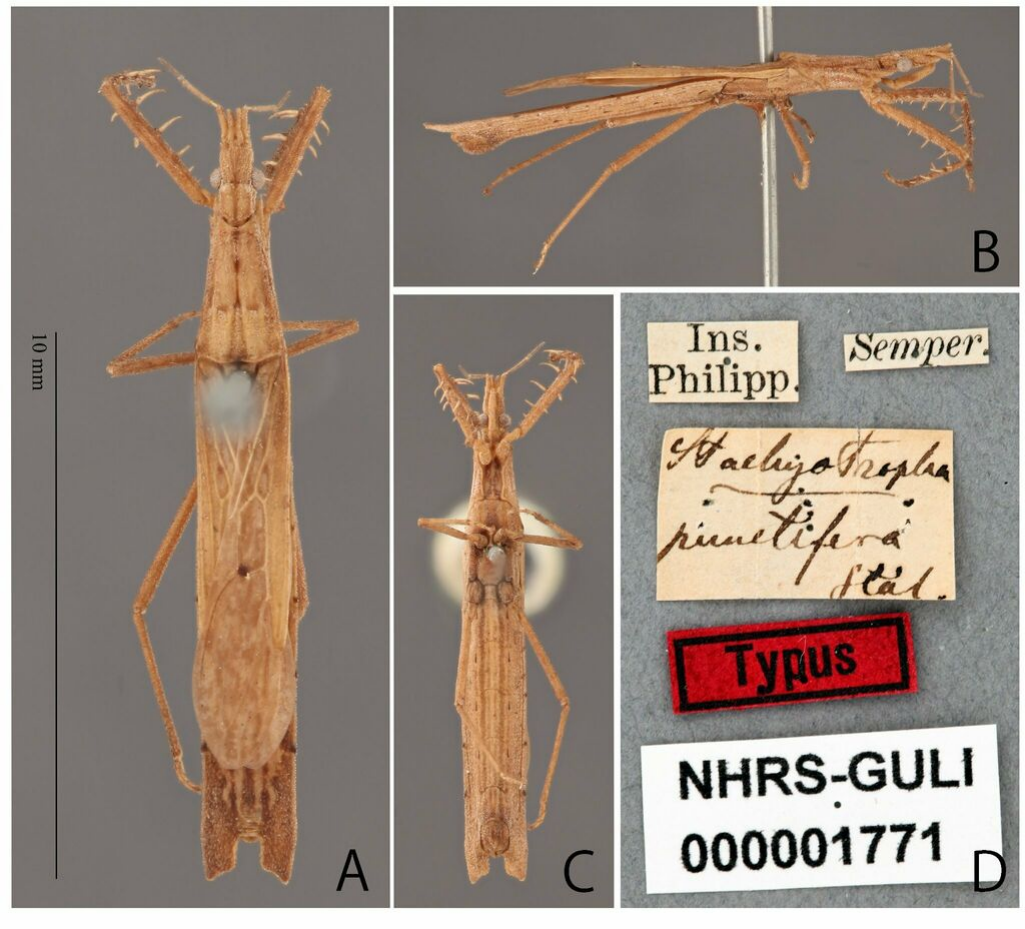
*Stachyotrophapunctifera* male, syntype deposited in NHRS, catalogue number NHRS-GU-LI000001771. **A** dorsal; **B** lateral; **C** ventral; **D** labels; Scale bar: 10 mm (A). Photos by Gunvi Lindberg, 2022; Naturhistoriska riksmuseet. Made available by the Swedish Museum of Natural History under Creative Commons Attribution 4.0 International Public License, CC-BY 4.0., https://creativecommons.org/licenses/by/4.0/.

**Figure 4.**
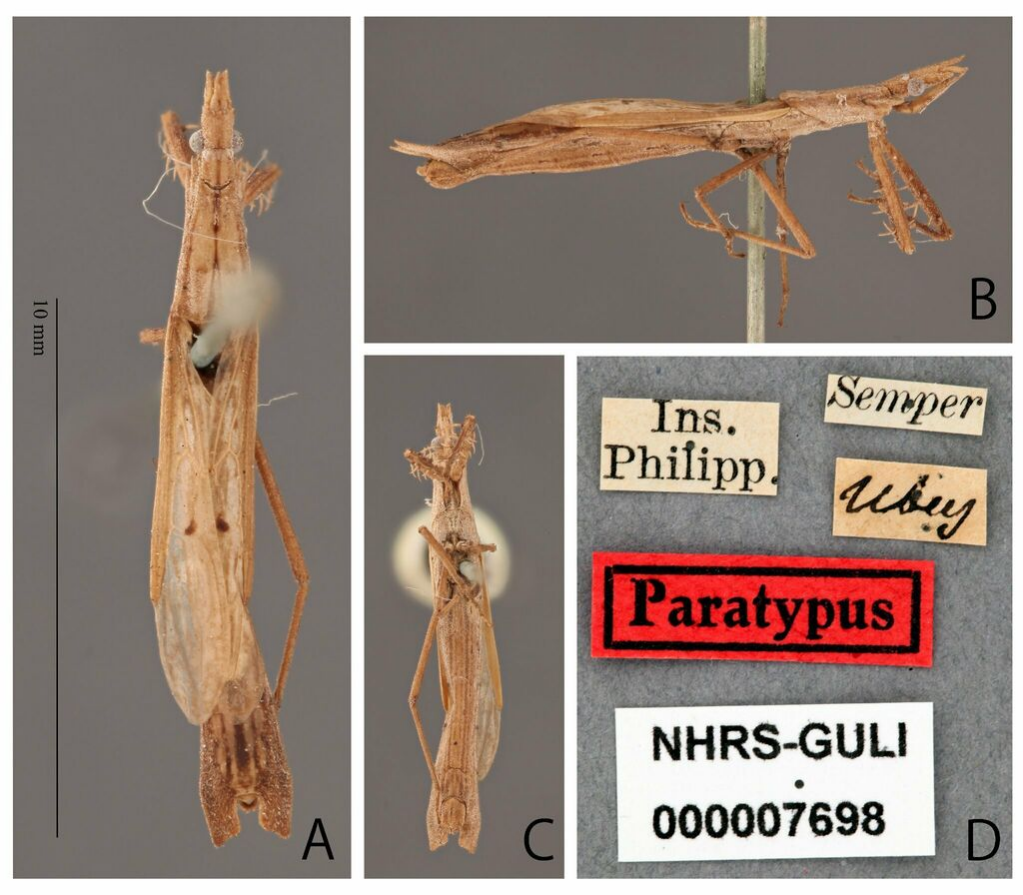
*Stachyotrophapunctifera*, male, syntype deposited in NHRS, catalogue number NHRS-GU-LI000007698. **A** dorsal; **B** lateral; **C** ventral; **D** labels; Scale bar: 10 mm (A). Photos by Gunvi Lindberg, 2022, Naturhistoriska riksmuseet. Made available by the Swedish Museum of Natural History under Creative Commons Attribution 4.0 International Public License, CC-BY 4.0., https://creativecommons.org/licenses/by/4.0/.

**Figure 5.**
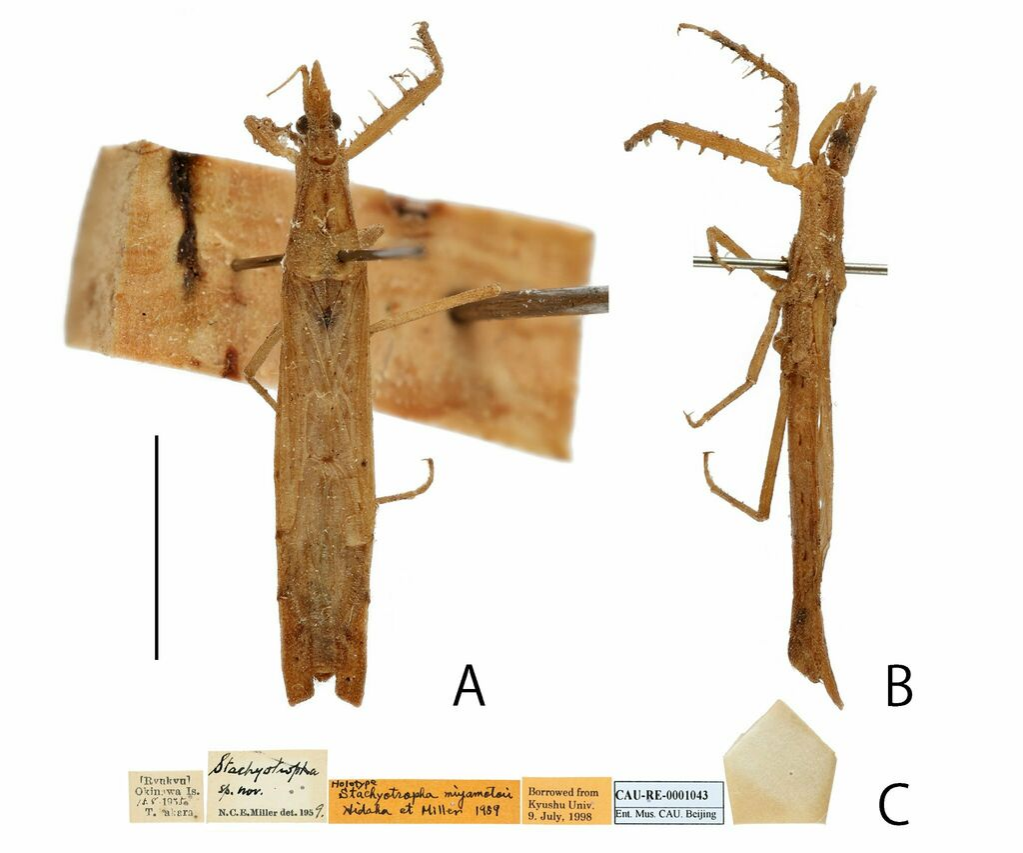
Photographs of holotype of *Stachyotrophamiyamotoi*: **A** dorsal; **B** lateral; **C** labels; Scale bar: 5 mm.

**Figure 6.**
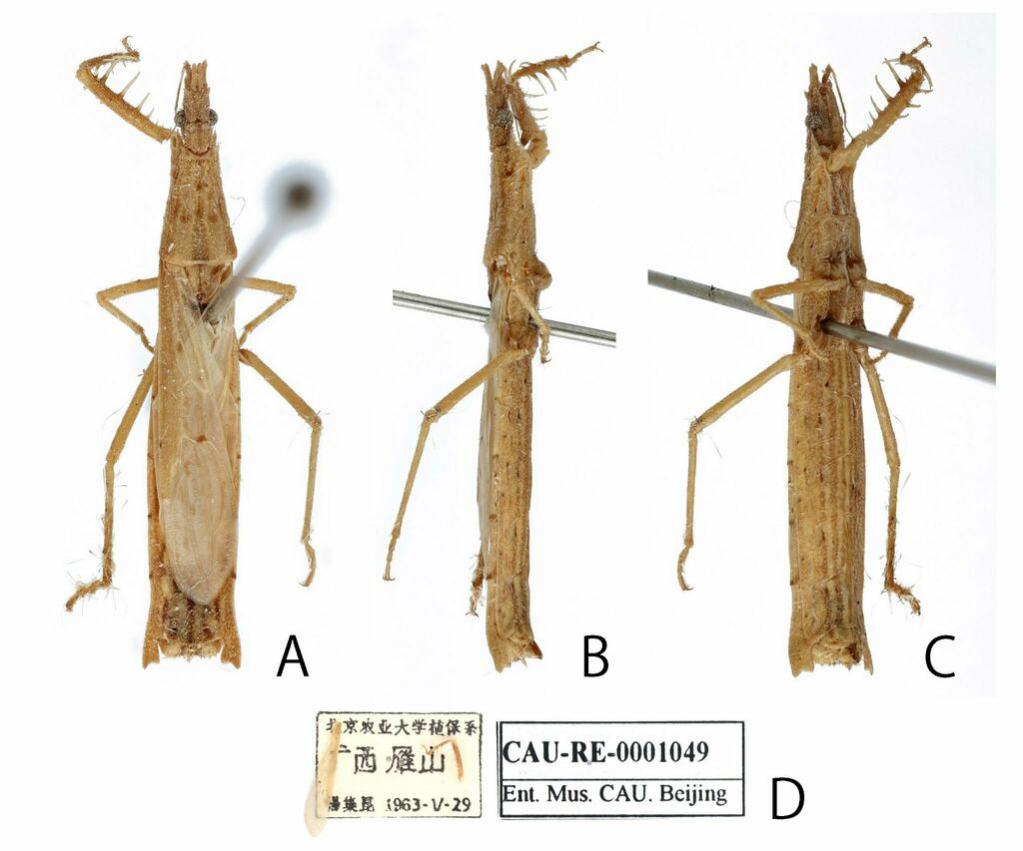
Photographs of a Chinese specimen of *Stachyotrophapunctifera*, female: **A** dorsal; **B** lateral; **C** ventral; **D** labels.

Based on the examination of the syntypes of *S.punctifera* (Figs 3, 4), the holotype of *S.miyamotoi* (Fig. 5) and several non-type specimens collected from China, Japan and the Philippines, we further evaluated and discussed the differences between *S.punctifera* and *S.miyamotoi*. The first four characters listed by Hidaka and Miller (1959) cannot be used to separate these two species because they were very similar between the examined specimens in this study. Additionally, the apical internal cell of the hemelytron varied significantly amongst our examined specimens. Furthermore, no morphological differences (including male paramere structures, but we could not compare genital structures) were found amongst these specimens. In Hidaka and Miller (1959), the description of *S.miyamotoi* was based on only one male specimen and they overlooked the fact that internal cells have a wide range of variation amongst individuals. This species has been known to be very rare. In fact, only one specimen was found in this study, which was collected from China (Fig. 6). Similarly, we consider that Hidaka and Miller (1959) also did not collect sufficient number of specimens for a proper comparative examination. In conclusion, as the comparison presented by Hidaka and Miller (1959) is inadequate and *S.miyamotoi* cannot be distinguished from *S.punctifera* by morphological characters, we propose that *S.miyamotoi* is a junior subjective synonym of *S.punctifera*.

## Supplementary Material

XML Treatment for Stachyotropha

XML Treatment for Stachyotropha punctifera
